# The New York Ambulance System

**Published:** 1882-02

**Authors:** 


THE NEW YORK AMBULANCE SYSTEM. — The New York ambulance system is undoubtedly the most perfect in the world.

It originated about ten years ago, the first one being used at Bellevue Hospital. Previous to that time what were known as "sick-wagons" were used, which were slow, lumbering affairs, not unlike an ordinary grocery wagon, and in marked contrast to the present brightly varnished, gilt-lettered ambulances which may be seen at almost any hour of the day dashing through the streets at what sometimes seems to be a perilous speed. The calls were also much longer in reaching the hospitals then than now, as the present perfect telegraph system, by which a call can be instantly sent from any police station to any of the larger hospitals, was not in use. If the case be an urgent one the call can be sent from the nearest fire alarm box, by which means valuable time is frequently saved. Under such circumstances it is called a "fire-call," and is transmitted to all of the hospitals, and the ambulance which first arrives claims the case. The ambulance surgeons and drivers are on duty both night and day, and seldom require more than two minutes to get in readiness for the start. A tourniquet, bandages, carron-oil and medicines useful in emergencies are always carried, and important services are frequently rendered before the hospital is reached. About two years ago an ordinance was passed, giving ambulances the right of way "against all persons, vehicles or animals," and since then but little obstruction has been met with, and the efficiency of the service consequently much increased. The introduction of ambulances was an important step in hospital management, and they certainly could not now be dispensed with.

